# Cytokines and Soluble Receptors in Breast Milk as Enhancers of Oral Tolerance Development

**DOI:** 10.3389/fimmu.2019.00016

**Published:** 2019-01-22

**Authors:** Bassel Dawod, Jean S. Marshall

**Affiliations:** ^1^Department of Pathology, Dalhousie University, Halifax, NS, Canada; ^2^Department of Microbiology and Immunology, Dalhousie University, Halifax, NS, Canada

**Keywords:** food allergy, sCD14, soluble CD14, TLR2, mucosal immunology, intestinal barrier function, regulatory T (Treg) cells

## Abstract

The postpartum period is an important window during which environmental factors can shape the life-long health of the infant. This time period often coincides with substantial milk consumption either in the form of breast milk or from cow's milk sources, such as infant formulas. Although breast milk is the most beneficial source of nutrients for infants during the first 6 months after birth, its role in regulating food allergy development, through regulation of oral tolerance, is still controversial. Breast milk contains several factors that can impact mucosal immune function, including immune cells, antibodies, microbiota, oligosaccharides, cytokines, and soluble receptors. However, there is considerable variation in the assessed levels of cytokines and soluble receptors between studies and across the lactation period. Most of these cytokines and soluble receptors are absent, or only found in limited quantities, in commercial baby formulas. Differences in content of these pluripotent factors, which impact on both the mother and the neonate, could contribute to the controversy surrounding the role of breast milk regulating oral tolerance. This review highlights current knowledge about the importance of cytokines and soluble receptors in breast milk on the development of oral tolerance and tolerance-relateddisorders. Understanding the mechanisms by which such milk components might promote oral tolerance could aid in the development of improved strategies for allergy prevention.

## Introduction

Oral tolerance is a state of immune non-responsiveness to antigens consumed by the oral route and derived from diet, environment, or gastrointestinal microbiota (1). Failure to develop proper oral tolerance, early in life, has been linked to several diseases, including food allergy, celiac disease, and inflammatory bowel diseases (IBD) (2). The incidence of allergic disease has been rising in recent decades, most notably in developed countries in association with environmental and lifestyle changes (3, 4). The concept of a “neonatal window of opportunity” has been proposed to describe the prenatal or perinatal period during which dietary and environmental factors can shape the development of the immune system and impact the susceptibility to immune-mediated diseases, including allergy (5). This period often coincides with substantial milk consumption either in the form of breast milk or infant formulas.

Breast milk is a valuable nutritional fluid rich with dietary antigens and immunomodulatory factors, including cytokines, soluble receptors, growth factors, short-chain fatty acids, vitamins, and microbiota, that are linked to the development of the neonatal gastrointestinal tract (GIT) and immune system. In this review, we will consider the role of milk components on the development of oral tolerance early in life with a particular emphasis on the cytokines and soluble receptors that could play a pivotal role in skewing the neonatal immune system toward either tolerogenic or allergic responses.

## Breastfeeding and Allergic Diseases

Breastfeeding is recommended by scientists, health organizations, and governments for all infants as a natural source of multiple factors that promote healthy immune responses, nutrition, protection against infection, and development (6). The World Health Organization recommends exclusive breastfeeding for 6 months before introducing solid foods (7). The impact of breastfeeding on the development of allergic diseases has been extensively studied, with conflicting results (8). Some have reported beneficial effects (9–15), while others have found no impact or increased risk of allergies among children that are breastfed, notably by atopic mothers (16–19). A randomized trial by Lucas et al. (15), found that feeding banked human milk to preterm infants reduced the risk of cow's milk allergy when compared with formula feeding. In contrast, a cohort study by Wetzig et al. (20), found that exclusive breastfeeding for more than 5 months was associated with increased early sensitization to eggs and atopic dermatitis. This variable effect of breastfeeding on the prevention of food allergy may be associated with differences in milk components related to ethnicity, diet, and other factors.

Breast milk composition is dynamic and changes dramatically over time to match the needs of the growing infant. For example, the protein concentration is about 1.4–1.6 g/dl in the colostrum and decreases to 0.7–0.8 g/dL after 6 months (21). The most common alternative for human milk is infant formula derived from cow's milk, which contains higher concentrations of protein and fat than breast milk (22). Breast milk is enriched with allergens that are ingested by the mother, such as β-lactoglobulin (23), ovalbumin (24), and peanut components (25). In a cohort study by Pitt et al. (26), the rates of peanut allergy were found to be significantly reduced among children whose mothers consumed peanuts while breastfeeding. A study by Grimshaw et al. (27) showed that infants who were diagnosed with food allergies at 2 years of age were more likely to have received solid foods at early ages (≤16 weeks of age) and less likely to be breastfed during the introduction of these foods. Furthermore, according to the Canadian Healthy Infant Longitudinal Development (CHILD) study, a delay in the introduction of food allergens, such as peanut, cow's milk, and eggs, can increase the incidence of food sensitization (28). Together, the transfer of food allergens in milk and the timing of solid food introduction relative to breast milk consumption appears to be critical for preventing allergic disease. The presence of immunomodulatory components in breast milk are thought to be critically important in regulating these processes.

## The Development of Oral Tolerance in Early Life

The intestinal barrier is exposed to a copious antigen burden. A properly functioning immune system must maintain tolerance to innocuous dietary, endogenous, and microbial antigens while responding to pathogenic insults. Immune tolerance to orally ingested antigens is characterized by decreased antigen-specific delayed-type hypersensitivity, T-cell proliferation, cytokine production, and reduced specific IgE (2). During the fetal period, the intestinal barrier is highly permeable, absorbing nutrients from the amniotic fluid (29). During the first week after birth, the permeability of the intestine rapidly decreases due to the maturation of the intercellular tight junctions between intestinal epithelial cells (IEC) (30). This process is accelerated in infants that ingest the colostrum while non-breastfed children experience a prolonged increased-permeability period (31, 32). Intestinal permeability also decreased faster in preterm infants (≤32 weeks of gestation) fed with breast milk rather than infant formula (33). Prolonged greater intestinal permeability could be linked to an increased incidence of atopic and infectious diseases in non-breastfed infants (34).

The GIT harbors a highly specialized immune system that includes gut-associated lymphoid tissues (GALT), such as Peyer's patches (PPs) and mesenteric lymph nodes (MLNs). These compartments harbor specialized antigen-presenting cells (APCs), including CX3CR1^+^ macrophages and CD103^+^ dendritic cells (DCs). Naïve T cells could be skewed toward different phenotypes based on their interaction with these APCs (35). CX3CR1^+^ macrophages can skew naïve T cells toward Th17 in response to microbial signals (36–38). In contrast, CD103^+^ DCs metabolize vitamin A to produce retinoic acid (RA), which along with TGF-β drive the conversion of naïve T cells into antigen-specific T regulatory cells (Tregs) and inhibit Th17 differentiation (39–41). Tregs enforce oral tolerance induction relevant to allergy via inhibition of allergen-specific Th2 responses and IgE class switching by B cells (42). The frequency of APCs in the intestine is dependant on the microbiota and the cytokine milieu (43, 44). Neonatal IECs have limited microbial communities and secrete low levels of cytokines and chemokines leading to a paucity of CD103^+^ tolerogenic DCs in the lamina propria (44). However, breast milk-derived mediators, including microbiota (e.g., *Bacteroides fragilis*), vitamin A and immune factors (such as TGF-β) compensate for this deficit and enhance the expansion of tolerogenic DCs (45–49).

Accordingly, the development of oral tolerance in children depends on dietary factors including those derived from maternal milk, which contribute to both immune regulation and maturation of the intestinal barrier. Defining the important regulatory factors in breast milk might expand our knowledge of the mechanisms involved in the development of food allergy.

## Breast Milk Cytokines and Soluble Receptors and Oral Tolerance

Over the past 20 years, multiple cytokines and immunomodulatory factors have been identified in breast milk. This list of mediators is increasing with advances in detection methods (Table 1). Many of these factors are derived from the epithelial cells of the mammary gland or from immune cells found in the milk (74) while others are transferred from the mother's circulation. Such breast milk components could impact the development of neonatal oral tolerance through both immune modulation and impacts on other systems such as epithelial barrier function or the intestinal microbiome. Particular challenges for research in this area are the variability in concentrations of immune factors in breast milk and their poorly defined ability to survive the infant's stomach and exert a biological effect in the GIT. Due to ethical limitations, most studies of the effect of breast milk immune factors on the host have been conducted either *in vitro* or *in vivo* using animal models. Through analysis of such studies, it is widely agreed that TGF-β, IL-10, IL-6, and sCD14, have a positive impact on tolerance development (75) while a number of other cytokines and soluble receptors are of potential importance. In addition to these factors, several chemokines, such as CXCL8, CCL2, CCL5, and CXCL10, as well as growth factors, such as EGF and IGF-(I and II), are detected in breast milk (76, 77), but are not the focus of this review.

**Table 1:** Concentrations of cytokines and soluble receptors in human colostrum and human milk.

	**Human colostrum (0–4 days)**	**References**	**Human milk (1–6 months)**	**References**
TGF-β1	140–3,300 pg/ml	(50, 51)	80–600 pg/ml	(50, 52)
TGF-β2	100–3,300 pg/ml	(50, 53)	800–5,300 pg/ml	(51, 54)
IL-1β	0.29–27.7 pg/ml	(51, 55)	0.028–23 pg/ml	(51, 52)
IL-4	1.6–172 pg/ml	(55, 56)	5.6–626.8 pg/ml	(54, 57)
IL-5	6.2–79 pg/ml	(54, 56)	6.2–142 pg/ml	(54, 56)
IL-6	7.3–80.6 pg/ml	(55, 58)	3.5–148.6 pg/ml	(51, 57)
IL-10	0–3,304 pg/ml	(59, 60)	0–246 pg/ml	(56, 59)
IL-12	3–310 pg/ml	(61, 62)	3–40 pg/ml	(61, 62)
IL-13	3.2–243 pg/ml	(54, 63)	3.2–264 pg/ml	(54, 56)
TNF	21.9–620 pg/ml	(64, 65)	4.4–58 pg/ml	(52, 66)
IFN-γ	2.5–708 pg/ml	(51, 56)	0.7–175 pg/ml	(51, 56)
G-CSF	4.38 pg/ml	(67)	4.2 pg/ml	(67)
GM-CSF	23.02 pg/ml	(67)	1.6 pg/ml	(67)
M-CSF	3,740–52,470 U/ml	(68)	1,150 U/ml	(68)
sTNF-R-I	3,703 pg/ml	(69)	1,732 pg/ml	(69)
sTNF-R-II	4,507 pg/ml	(69)	931 pg/ml	(69)
sIL-6R	12,761 pg/ml	(69)	2,436 pg/ml	(69)
sCD14	77.9–88.8 μg/ml	(70)	7–25 μg/ml	(71, 72)
sTLR2^*^	+	(73)	+	(73)

* *Concentration of sTLR2 in human milk is not available*.

## Cytokines

**Figure 1:**
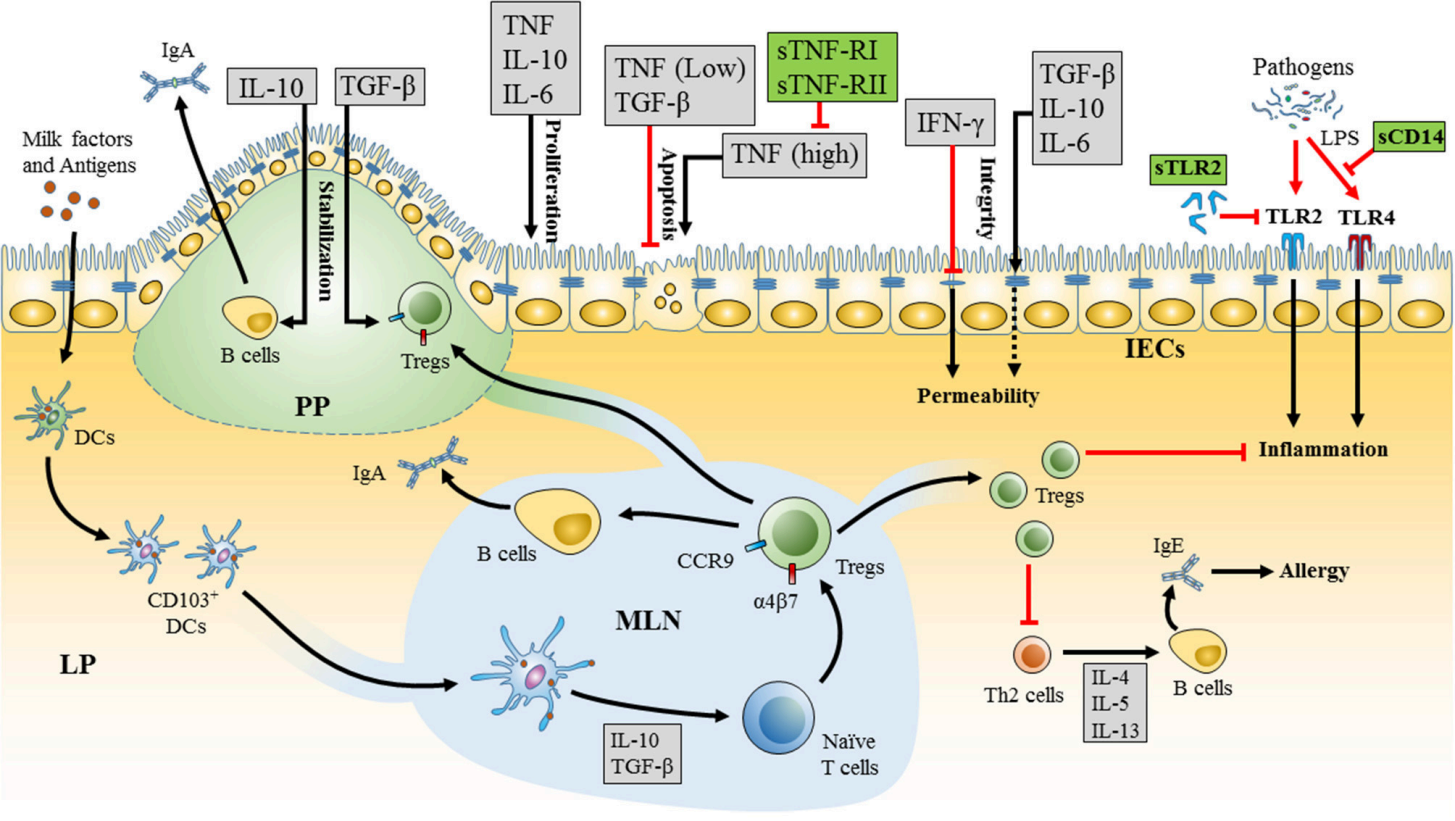
Mechanism of milk cytokines and soluble receptors in promoting oral tolerance in the neonatal intestine. Milk factors enhance the development of tolerogenic dendritic cells (DCs) (CD103^+^) in the neonatal gastrointestinal tract (GIT). These DCs sample milk antigens and migrate to the mesenteric lymph node (MLN). Tolerogenic DCs in the MLN drive the differentiation of naïve T cells into T regulatory cells (Tregs) and the expression of α4β7 integrins and CCR9 receptors that are essential for homing of Tregs to the lamina propria (LP) and Peyer's patches (PP). Tregs enhance oral tolerance by inhibiting inflammation and T helper 2 (Th2) responses and induce secretion of IgA from B cells. Milk derived cytokines (gray boxes) and soluble receptors (green boxes) form a network of immunomodulators that interact together and impact oral tolerance via a variety of mechanisms. Milk cytokines, including as TGF-β, IL-10, IL-6, TNF, and IFN-γ, affect the integrity, proliferation, and apoptosis of intestinal epithelial cells (IECs). High levels of cytokines in breast milk could also have adverse effects, such as high concentrations of TNF that could be seen in mastitis and induce apoptosis in the IECs. The effects of TNF can be attenuated via the corresponding soluble receptors, sTNF-R-I, and sTNF-R-II that are found in breast milk. Furthermore, soluble toll-like receptor 2 (sTLR2) and soluble CD14 (sCD14) in breast milk can modulate the inflammatory response toward pathogens in the neonate's GIT by regulating TLR2 and TLR4 mediated cell activation, respectively.

Cytokines detected in breast milk, include TGF-β, IL-10, IL-6, IL-1β, TNF, IFN-γ, IL-4, IL-5, IL-12, IL-13, G-CSF, GM-CSF, and M-CSF (Table 1) (63, 67, 68, 78, 79). Many of these cytokines have the potential to alter oral tolerance via their impact on the development of the infant's immune system and GIT (Figure 1). They may also impact the function of the mammary gland in the mother. Several factors might further influence the concentration of cytokines in breast milk. For example, subclinical mastitis, a local inflammation in the mammary gland observed in 23% of nursing mothers, induces considerable changes in milk pro-inflammatory cytokines that might affect infants (80).

The most abundant cytokines in breast milk are TGF-β family members, including TGF-β1 and TGF-β2. The concentration of TGF-β differs dramatically through the lactation period and between individual mothers, with TGF-β2 being more abundant in breast milk and TGF-β1 in the serum while both are relatively scarce in infant formula (54, 81, 82). The majority of TGF-β1 and TGF-β2 in breast milk exists in a latent form that gets activated by the gastric acid in an infant's stomach (83). Furthermore, CD103^+^ DCs have the ability to activate latent TGF-β, which is important for these DCs to induce Tregs (84).

TGF-β has several anti-inflammatory roles, inhibiting naïve T cells from differentiation into Th1 and Th2 subtypes and thereby suppressing Th1/Th2 responses (85, 86). TGF-β also fosters stabilization of FOXP3 expression and maintains the differentiation of Tregs (87, 88). The roles for TGF-β in the GIT are multifaceted and include enhancing oral tolerance (89), promoting intestinal integrity (90), stimulating IgA class-switching in B cells (91), promoting colonization and increased abundance of microbiota (92), and regulating inflammatory responses (85, 86). According to a systemic review by Oddy et al. (93), high levels of TGF-β1 and TGF-β2 in breast milk were inversely correlated with the incidence of allergic diseases in childhood. Furthermore, the levels of TGF-β were higher in maternal colostrum of infants who developed post-weaning atopy compared with those with pre-weaning atopy (50). Furthermore, levels of TGF-β1 were significantly lower in the breast milk of allergic mothers compared to non-allergic mothers, potentially linked to increased symptoms of atopic dermatitis in infants born to allergic mothers (59). Although TGF-β can induce pathogenic Th17 responses in the presence of IL-6, the production of RA from CD103^+^ DCs in the intestine is thought to antagonize and override IL-6-driven induction of Th17 and promote Treg differentiation (40).

IL-10 is an important anti-inflammatory cytokine detected in both the breast milk whey fraction and fat. Breast milk derived IL-10 has a molecular weight >80 kD, higher than that of IL-10 in serum, suggesting that it might be bound to other molecules or post-transcriptionally modified (60). The bioactivity of IL-10 in breast milk has been confirmed (51). IL-10 increases the survival and expansion of B cells, inhibits Th1 responses and downregulates major histocompatibility complex-II expression on monocytes, thus, limiting their antigen presenting cell function (94). IL-10 has been heavily implicated in the regulation of intestinal inflammation and regulating responses to the microbiome.

IL-6 is a pleiotropic cytokine reported to have both pro-inflammatory (95) and anti-inflammatory (96) impacts with a key role in the regulation of the acute phase response which both enhances innate anti-bacterial host defense and limits some of the negative impacts of inflammation. IL-6 is also an important regulator of mucous production by goblet cells (97). It has been detected in the whey portion of breast milk in both high molecular weight ≥100 kD and 25–30 kD isoforms and at relatively consistent levels in breast milk for the first 3 months post-partum (98, 99). This cytokine has been linked to the production of IgA in the neonatal intestine by inducing follicular T helper cells in the germinal centers of PP (98). It also stimulates the mammary epithelium to transport more IgA into milk (100). The levels of IgA in breast milk are highly correlated with the concentrations of TGF-β, IL-10, and IL-6 in breast milk (54). High levels of IgA in breast milk have been reported to be protective against allergic disease development, including cow's milk allergy (24, 100).

IL-1β was probably the first cytokine to be quantified in breast milk using radioimmunoassay (RIA). Munoz et al. (101) reported that IL-1β was present in high concentrations in the colostrum and day 7 milk, however, more modest levels have been reported in more recent studies (51, 78, 102). Although IL-1β has been shown to attenuate skewing of T cells toward Tregs, Järvinen et al. (100) have shown that IL-1β together with IL-6, IL-10, and TGF-βl in breast milk are associated with enhanced tolerance to cow's milk. However, the impact of breast milk-derived IL-1β on tolerance development in neonates is still not clear, as both the cytokine and its natural antagonists, such as IL-1 receptor antagonist, are observed together in the milk.

*In vitro* and *in vivo* animal studies have suggested an important role for milk-derived cytokines on intestinal epithelial proliferation and repair. These activities are essential for maturation and healing of the GIT and involve milk derived cytokines such as TNF (103), IL-10 (104), and IL-6 (105). In addition, TNF and TGF-βl usually have an anti-apoptotic effect on IECs (103, 106), although very high concentrations of TNF will induce apoptosis (107). Intestinal permeability, which is a crucial factor in the regulation of oral tolerance, could be substantially altered by breast milk cytokines. *In vitro* experiments suggest that IL-10 enhances intestinal integrity and compromises the barrier disrupting effect of IFN-γ, a process confirmed by severe chemical-induced colitis and observations of increased intestinal permeability in IL-10 receptor 1 null mice (108). A study by Kuhn et al. (109) has shown a decrease in the expression of epithelial barrier proteins and a thinner mucus layer in the intestines of IL-6^−/−^ mice, suggesting a role of IL-6 in intestinal integrity. In addition, milk cytokines could also impact the mammary gland itself. For example, TNF is an important regulator of the development and branching of glands in the breast (110) such factors could impact both the available supply and constituents of breast milk.

The extent to which breast milk-derived cytokines exert their effects on the neonatal GIT also depends on several neonatal factors. Their concentrations in milk vary dramatically during the lactation period and are often higher in the colostrum (76). The ability of cytokines to retain bioactivity after passage through the infant's stomach is also critical. The pH in the neonatal stomach is higher than in adults (pH 3–5), which might allow more cytokines to exert biological effects and help compensate for the paucity of cytokine responses in neonates (111). Other factors might also impact the efficacy of milk-derived cytokines including the existence of soluble receptors or receptor antagonists in breast milk or the neonatal GIT, which might either regulate binding of the cytokines to their receptors or compete with them (69).

## Soluble Receptors

Soluble receptors are thought to have immunoregulatory roles in many biological fluids, including breast milk. They regulate signaling of milk-borne cytokines and innate immune stimulators through membrane-bound receptors in the neonates (Figure 1). Breast milk contains several soluble cytokine receptors, such as sIL-6R and sTNF-RI and sTNF-RII, receptor antagonists, such as IL-1RA, and soluble innate immune receptors, such as sCD14 and sTLR2 (Table 1). These receptors might in some circumstances be bound to their ligands or carrier proteins, which could explain the larger observed molecular weight of some cytokines in milk (≥100 kD and 25–30 kD for IL-6, from 80 to 195 kD for TNF, and >80 kD for IL-10) (69). However, this issue has not been well-studied.

Soluble receptors for classical inflammatory cytokines are found in breast milk throughout lactation. The levels of sIL-6R are low under normal conditions in both colostrum and mature milk and its affinity to IL-6 is also low (69, 112). The exact role of this receptor in breast milk is not clear yet, however, *in vivo* experiments have shown an augmentation of IL-6 function by sIL-6R (113).

IL-1RA is detected in human colostrum and milk in amounts higher than serum. It binds to the IL-1 receptor due to homology with IL-1α and IL-1β (69, 114). However, it is considered an antagonist as it competes with IL-1α/IL-1β for receptor binding and thus regulates their effects (69, 114, 115). The importance of IL-1RA in milk has not been well-studied, but it likely limits the inflammatory response in the neonatal GIT. The two soluble receptor forms of the TNF receptors are sTNF-RI and sTNF-RII. These been reported in both the human colostrum and milk and shown to modulate the effect of TNF on its receptor. Only a small fraction of the TNF in breast milk is free to activate cells while the majority is speculated to be neutralized by the soluble receptors (69). High levels of TNF have been detected in milk from mothers with mastitis; however, this was accompanied by elevated levels of sTNF-RII and IL-1RA, which might protect nursing infants from high pro-inflammatory cytokine levels in the context of such breast infections (116).

Soluble forms of innate immune receptors, CD14 and TLR2, have also been detected in breast milk (117). A single (48 kD) form of sCD14 has been observed in human milk, whereas sTLR2 is detected in six isoforms (ranging from 20 to 85 kD) (73, 118). There is substantial evidence that the responsiveness of TLRs to their ligands, such as the lipopolysaccharides (LPS) and bacterial lipopeptides in the neonatal intestine, is regulated by sTLRs and sCD14 leading to the inhibition of potentially damaging responses (119) allowing for more efficient development of tolerance to commensal microbiota. CD14 is a co-receptor for both TLR2 and TLR4 and facilitates recognition of their ligands (120). The interaction between sCD14 and sTLR2 in breast milk increases the binding capacity of sTLR2 to bacterial products, such as the peptidoglycan of Gram-positive bacteria (121). Furthermore, sCD14 can complex with LPS and limits LPS-mediated cellular stimulation (118, 122). The role of TLR2 in oral tolerance is still not clear as signaling via this receptor differs between commensal and pathogenic bacteria (123). Our group has shown that TLR2 activators in food might skew the immune system toward an allergic response by inhibiting oral tolerance development (124). In contrast, *B. fragilis*, that contains polysaccharide A signals via TLR2 on Tregs leading to suppression of Th17 response and enhanced colonization of this bacteria in the intestine (123). Therefore, establishment or disruption of tolerance via TLR2 might require the involvement of other microenvironmental ligands and/or receptors and be highly dependent on intestinal location. sTLR2 in breast milk has also been implicated in the prevention of HIV infection and inhibition of inflammation (125) although the mechanisms whereby this occurs are not well-understood. Improved intestinal barrier function or altered populations of, or receptor expression by local immune effector cells may contribute to altered vulnerability to infection. Little work has been done examining either the role of sTLR2 in oral tolerance or its impact on the developing microbiota in the neonate. Several further soluble receptors that exist in serum, saliva, or urine, including sTLR4, sIL-4-R, sIL-5-R, sIFN-γ-R, sTGF-β-R, sGM-CSF-R (126), might be of additional potential importance in regulating the impact of milk-borne cytokines, however, these receptors have not been well-studied and defined in breast milk.

## Conclusion

Breast milk contains a network of immune mediators, including several cytokines and soluble receptors that have not been well-studied in the development of oral tolerance in neonates. Strong evidence suggests a critical role for breast milk-derived immune mediators in preventing the development of allergic diseases, in part through modulation of the neonatal immune system and GIT maturation. Such mediators include soluble receptors and receptor antagonists that can minimize the adverse effects of such cytokines and pattern recognition receptors in the neonates GIT and developing the mucosal immune system. Differences in results from studies examining the presence and concentrations of cytokines and soluble receptors in breast milk may relate to the studied populations, collection time, sample storage, and methods of detection, as reviewed by Agarwal et al. (76). Much further work is needed to determine the extent to which milk derived cytokines and soluble receptors influence the development of oral tolerance and the subsequent expression of allergic disease. Breast milk dependent early life immune regulation, while challenging to study, provides important opportunities for immune interventions with long-lasting health impacts.

## Author Contributions

BD completed the literature search, wrote the first draft of the article, and prepared the figures and tables. JM provided the initial article concept edited and further contributed text to the manuscript and provided advice on review preparation and style, and input into figure and table.

### Conflict of Interest Statement

The authors declare that the research was conducted in the absence of any commercial or financial relationships that could be construed as a potential conflict of interest.
